# Comparative Characterization of Hemp Seed Cakes from Dehulled and Hulled *Cannabis sativa* L. var. oleifera cv. ‘Henola’: Nutritional, Functional, and Storage Stability Insights

**DOI:** 10.3390/foods14091605

**Published:** 2025-05-01

**Authors:** Krystian Ambroziak, Anna Wenda-Piesik

**Affiliations:** Department of Agronomics and Food Processing, Faculty of Agriculture and Biotechnology, Bydgoszcz University of Science and Technology, Al. Kaliskiego 7, 85-796 Bydgoszcz, Poland

**Keywords:** hempseed cake shelf life, dehulling, cold pressing, protein content, dietary fiber, antinutritional compounds, oxidative stability, microbiological quality, sensory evaluation

## Abstract

This study investigated the nutritional composition, antinutritional factors, oxidative stability, microbiological safety, and sensory characteristics of hempseed cake (HC) derived from *Cannabis sativa* L. cv. ‘Henola’. The effects of dehulling and storage (1, 3, and 6 months) on dehulled (DHC) and hulled (HHC) hemp cake were systematically assessed. DHC exhibited significantly higher crude protein (up to 42.2%) and residual oil content (up to 37.5%), while HHC was richer in dietary fiber (up to 41.3%) and total carbohydrates (up to 48.2%). Despite comparable PUFA contents (63–72%) and favorable n-6/n-3 ratios (~3.1:1), DHC showed greater energy concentration and reduced levels of indigestible carbohydrates and phytates. Oxidative stability tests revealed increased acid and peroxide values in both HHC and DHC after six months, indicating quality deterioration (Totox index > 15). Microbiological analyses confirmed hygienic safety across all samples, with slightly higher microbial counts in HHC linked to hull-associated contamination. Sensory evaluations revealed stable color, odor, and texture during storage, with DHC rated more aromatic. These findings confirm that processing conditions—particularly dehulling—strongly affect the functional and nutritional profile of hempseed by-products. DHC emerges as a promising, shelf-stable, protein-rich ingredient for functional food and feed applications.

## 1. Introduction

Hemp (*Cannabis sativa* L.), a member of the Cannabaceae family, originated in Central Asia and southeastern Europe [1]. The species is divided into two main subspecies—*sativa* and *indica*—and possibly a third taxon, *C. ruderalis* [2]. Historically, hempseed oil was used for lamps, soap, varnish, paint, and as a feed supplement [3,4,5,6]. Today, hemp is recognized as an industrial crop with significant nutritional potential [7]. Rich in protein, essential amino acids, fiber, vitamin E, and minerals, hemp seeds enhance both human diets and animal feeds [8,9,10]. However, they are relatively low in lysine and, to a lesser extent, leucine and tryptophan [11]. Protein digestibility improves with dehulling, reaching up to 97.5% in peeled seeds compared to 85% in whole seeds [5,11,12,13,14]. The primary proteins, edestin and albumin, are easily digestible and nutritionally comparable to those in other high-quality plant sources [15,16,17]. Hemp peptides also show antioxidant and anti-inflammatory properties [18,19,20,21].

The oil content in hemp seeds exceeds 30%, with high levels of linoleic (omega-6) and alpha-linolenic (omega-3) acids, typically in a favorable 3:1 ratio [22,23,24]. This lipid profile, with low saturated fats and a high PUFA content, is beneficial for cardiovascular health [20,25,26,27]. After cold pressing, partially defatted hemp flour retains 13–15% oil, depending on the cultivar [28].

In response to the growing global demand for plant-based protein and fiber sources, hemp-derived ingredients are increasingly explored for food and feed applications. The present study focuses on a Polish cultivar ‘Henola’, known for its high yield and oil content [29]. It aims to (i) compare the nutritional and functional value of hemp cakes from hulled and dehulled seeds, (ii) assess the effect of processing on antinutritional factors, and (iii) evaluate the shelf life and sensory stability of these by-products. The hypothesis is that hemp cakes can offer nutritionally valuable, microbiologically safe ingredients for use over extended storage periods. To our knowledge, this is the first comprehensive study to evaluate the long-term storage stability and nutritional dynamics of dehulled and hulled hemp cakes from the ‘Henola’ variety under real storage conditions.

## 2. Materials and Methods

### 2.1. Characteristics of ‘Henola’ Hemp

Non-narcotic, monoecious oil variety ‘Henola’, bred by the Institute of Fibrous and Medicinal Plants of the Polish Research Institute, number R2908, was chosen for this study. This variety is recommended for the cultivation of hemp for seeds. It is characterized by producing at least twice the yield compared to other varieties available on the market (0.8–2.0 tons per hectare for industrial plantations (Table 1). ‘Henola’ characteristics include low height < 2 m and a 3-week shorter vegetation period as compared to typical fiber varieties [29].

### 2.2. Agrotechnics

In the 2023 season, hemp sowing was completed by utilizing a traditional grain seeder, row spacing of 12 cm, and sowing depth of 3 cm; seeds were sown in the amount of 60 kg ha^−1^. No herbicides or fertilization were used, due to the conversion of experimental plantations to an ecological system. Hemp seeds were harvested with a combine harvester at the stage of full seed maturity. Prior to harvest, seed moisture amounted to 26%.

### 2.3. Laboratory Tests on Hemp Seeds

The size of hemp seeds was determined by dividing them into fractions using the sieve method, of >2.8 mm, 2.5–2.8 mm, and 2.2–2.5 mm, which determined their usage either for hulling or direct pressing without hulling. The buttock with the smallest fraction (below 2.2 mm) was not subjected to further testing. Hemp seeds were tested for oil, total protein, and fiber using near-infrared reflectance spectroscopy (NIRs) (Foss Infratec NOVA, Hillerød, Denmark).

### 2.4. Method of Hulling and Processing Hemp Seeds

Hemp nuts designated for seed coat removal underwent initial cleaning utilizing an industrial sieve and fan windmill (Sitono CZ Maior, Staroszki, Poland). The dehulling process took place on a test stand created exclusively for this purpose and consisted of 3 stages. The first stage involved a Hemp Seed Dehulling Machine GG-1 (Longer Machinery, Zhenghou, Henan, China), which effectively split the seed husk and partially removed it using an integrated aspiration system. In the second stage, cracked seeds were redirected to a mechanical mixer (Grutech GTH-MBM SD, Janowo, Poland) to separate the seed husks from the kernels using a spiral ribbon. The resulting mixture was then transferred to a machine (Pfeuffer GmbH MLN, Kitzingen, Germany) with a built-in air aspiration system used for suctioning out the husk and, therefore, to complete the final stage of the process (separation of the husk from the seminal nuclei). Dehulled hemp seeds were used to produce hemp cake. A snail press (Miramar Sp. Z o.o. M-22, Nowa Wieś Poland) with adaptable components for seeds with different degrees of hardness and variable compression ratios, shaft speed ranges, and pressing process temperatures was used for this purpose.

The research involved two products: dehulled hemp cake (DHC) and hulled hemp cake (HHC), both produced through cold pressing of oil at 55 °C. In subsequent research stages, DHC was subjected to two additional production variants: double cold pressing of oil at 55 °C and single hot pressing at 90 °C. These additional DHC variants were not tested for shelf life but were used to compare the oil content in the cake and tested for the presence of antinutritional substances.

The extracted product obtained through pressing was hemp cake in the form of pellets. Prior to laboratory testing, the samples were ground and placed in dark, light-restricted packaging with double zipper closure.

### 2.5. Analytical Methods for Testing Hemp Cakes

#### 2.5.1. NIRs and Laboratory Methods

Hemp cakes obtained in the pressing process were ground (Foss Knifetec KN 295 mill, Hillerød, Denmark), designed for fat samples, and cooled with water. The NIRs method was initially selected on Foss analyzers (DA1650, Hillerød, Denmark), and then on Bruker MPA II (Bruker Optics GmbH & Co, Ettlingen, Germany), according to PN EN ISO 12099:2017. The content of crude protein (CP), according to PN-EN ISO2043:2007, crude fiber (CF), according to PN-EN ISO 6865, and oil content (OC), according to PN-EN ISO 734-1:2007, were investigated. The same samples were studied using wet reference methods, with the water and ash content (%) measured by weight methods. Protein was determined using the Kjeldahl method on a Foss distiller (Kjeltec 9, Hillerød, Denmark), in accordance with AN 5511, v.3. Fiber was determined using an enzymatic-gravimetric analysis (Foss, Fibertec™ 8000, Hillerød, Denmark) in accordance with AN 3440, v. 2. Total fat extraction was carried out using a Foss extraction system (Soxtec™ 8000, Hillerød, Denmark) and a hydrolysis system (Hydrotec™ 8000, Hillerød Denmark). Fat extraction was carried out according to Randall’s modification using the Soxhlet method. Analyses were performed in accordance with AN 320. Fatty acid (FA) profiles were assessed with GC using official ISO protocols: SFA, MUFA, PUFA, n-3 (ALA, EPA, DHA, ETE, DPA), n-6 (LA, GLA, ARA, DGLA), (C18:2w6) cis-linoleic acid (LA), (C18:3w3) alpha-linolenic acid (ALA), and (cis-9, cis-12, cis-15 alpha-linolenic acid), according to PN-EN ISO 12966-1:2015-01 + AC:2015-06 + PN-EN ISO 12966-2:2017-05 in g100 g^−1^ oil.

Cake samples tested by NIRs and wet reference methods were further analyzed for their energy value (EV) in kcal100 g^−^^1^, according to Regulation (EU) No. 1169/2011 (OJ L 304, 22 November 2011, as amended). The total carbohydrate (TOT Carb in %) and digestive carbohydrates (Dig Carb in %) were calculated, and total sugars (TOT Sugar %) were measured according to Regulation (EC) No. 152/2009 of 27 January 2009 (OJ EU L 54/1 of 26 February 2009). The dietary fiber (DF), non-starch polysaccharide (NSP), Klason lignin (KL), uronic acid (UA), raffinose family oligosaccharides (RFOs), phytic acid (PA), and total phenolic content (TPC) were laboratory tested.

#### 2.5.2. Dietary Fiber

DF was determined using the enzymatic-gravimetric (AOAC 985.29) method as the sum of NSP, KL, UA, and oligosaccharides.

#### 2.5.3. Non-Starch Polysaccharides

The content of NSP was determined by gas chromatography according to Englyst and Cummingst [30], the AACC standard procedure 32-25, and AOAC 994.13 [31]. The T-NSP is the sum of sugars: arabinose, xylose, mannose, galactose, and glucose (Approved Methods of the AACC, 2003; AOAC, 1990) [32]. This analysis allows the separation of NSP into two fractions: soluble and insoluble, and the determination of the polysaccharide composition in both fractions. The content of arabinoxylans in each fraction is calculated as the sum of arabinose and xylose. In the first stage, enzymatic hydrolysis of starch is carried out using alpha-amylase and amyloglucosidase enzymes, and then the samples are centrifuged and separated using 96% ethyl alcohol into soluble (supernatant) and insoluble (pellet) fractions. Each fraction is hydrolyzed using 1 M sulfuric acid (100 °C, 2 h) to monosaccharides and then converted to volatile alditol acetates. The samples prepared in this way are separated on a Clarus 500 gas chromatograph (PerkinElmer, Shelton, Connecticut, USA) equipped with an Rtx-225 quartz capillary column (0.53 × 30 m), autosampler, split injector, and flame ionization detector (FID). Carrier gas for analyses: helium. The separation is carried out at 225 °C, with the injector and detector temperature at 275 °C.

#### 2.5.4. Klason Lignin

The KL content was determined by a gravimetric method (AACC 32-25) [33]. The ground sample was incubated with 72% sulfuric acid at 30 °C for 60 min, and then deionized water was added and incubated at 100 °C for 180 min. After cooling the contents of the tubes, the insoluble fraction was recovered by filtration, washed with water, 80% ethyl alcohol, and acetone, and then dried (105 °C, 16 h) and ashed (550 °C, 5 h). The lignin content was calculated from the weight loss after ashing of the dried material [33].

#### 2.5.5. Uronic Acid

The UA content was determined by the colorimetric method according to Scott [34] and Englyst et al. [35]. Galacturonic acid was used as a standard. To the hydrolysates from the acid hydrolysis of NSP from the soluble and insoluble fractions, 3% boric acid solution and 2% sodium chloride were added. Then, concentrated sulfuric acid was added and heated at 70 °C for 40 min. After cooling, a solution of 3,5-dimethylphenol in acetic acid was added and mixed, and the absorbance was measured at 400 and 450 nm.

#### 2.5.6. Raffinose Family Oligosaccharides

The content of RFO was determined by gas chromatography according to Lahuta [36]. Oligosaccharides were extracted with 50% ethyl alcohol and then converted into volatile derivatives using a mixture of trimethylimidazole and pyridine (1:1, *v*/*v*). The derivatives were separated on an Rtx-1 capillary quartz column (0.25 mm × 15 m) using a Clarus 600 gas chromatograph (Perkin Elmer). Oligosaccharides were calculated as the sum of raffinose, stachyose, and verbascose.

#### 2.5.7. Phytic Acid

Phytic acid (PA) was determined by the colorimetric method according to Haug and Lantzsch [37]. The sample with 0.2 N HCl added was extracted for 3 h at room temperature and then centrifuged. Ammonium and iron sulfate were added to the obtained filtrate and boiled for 30 min. After this time, the samples were cooled and centrifuged for 30 min at 6 °C. Then, bipyridine solution was added to the obtained supernatant and, after 10 min, the absorbance was measured at a wavelength of 519 nm.

#### 2.5.8. Total Phenolic Content

The total phenolic content (TPC) was assessed using the colorimetric Folin–Ciocalteu method as described by Shahidi and Naczk [38]. Samples were extracted twice with 80% methanol at room temperature for 2 h. After centrifugation, the combined supernatants were reacted with Folin–Ciocalteu reagent, followed by sodium carbonate addition to establish alkaline conditions. Absorbance was measured at 750 nm after 100 min of incubation in the dark. Gallic acid served as the standard, and the results were expressed as mg gallic acid equivalents (GAEs) per gram of dry mass. Analyses were conducted in duplicate, and means were accepted when replicate differences were below 4%.

The results based on NIRs technology were used for device calibration, aiming to save time and costs in future laboratory evaluations for hemp cakes. Spectra collected in the continuous production process were invaluable in validating the developed research method.

#### 2.5.9. Storage Tests

The storage trials consisted of testing the nutritional value and organoleptic properties of the sample at three different times, i.e., after 1, 3, and 6 months from production and packaging. The acid value (AV) in mg KOH g^−1^ (PB-PAZ/FS-24 v. 01, 22.02.2021 r.), peroxide value (PV) in meq O_2_ kg^−1^ (PN-EN ISO 27107:2012), and anisidine value (ANV) (PN-EN ISO 6885:2016-04) were measured, while the Totox index was calculated by the formula 2PV + ANV, for both oil and cakes in identical temperature and time conditions. The microbiological status of cakes was measured by counting (in cfu g^−1^) mesophilic aerobic microorganisms (MAMs) at 30 °C, yeasts and molds (Y&M), and coagulase-positive staphylococci (CPS) of *Staphylococcus aureus*, *Enterobacteriaceae*, and *Salmonella* spp., according to the following methods: PN-EN ISO 4833-1:2013-12, PN-ISO 4832:2007, PN-ISO 7954:1999 (W), PN-EN ISO 21528-02:2017-08, PN-EN ISO 6888-2:2022-03, and PN-EN ISO 6579-1:2017-04 + A1:2020-09.

The same three-person panel conducted a sensory analysis of the hemp product’s characteristics. General appearance and consistency (PB-PAZ/FS-33 v. 01, 4 March 2021), color and smell (PN-R-74013:2012) were assessed and scored on nominal scales. The descriptive data for each feature were calculated using a sign test to verify the hypothesis of no variation in color, appearance, and odor over the period of 6 months. Data from the test protocols supported the patent application.

### 2.6. Statistics

Nutrient content data, with threefold replications for each research object (HHC/DHC) in each month, were analyzed for a normal distribution using the Shapiro–Wilk *W* test. The normalized data were subjected to calculations using the *t*-Student test. The results are presented as mean ± standard error (s_e_). The contents of nutrients and antinutrients were subjected to one-way ANOVA in order to verify the null hypothesis assuming no differences between the experimental oil extraction process. For this purpose, the ANOVA test, *F*, was used at the significance level of *p* = 0.05. In a situation where the significance of the extraction process effect was proven, a comparison of the object means was performed using the Tukey *HSD* test, for *p* = 0.05. The non-parametric sign test was calculated to verify the hypothesis regarding the changes in organoleptic features on nominal scales (smell, color, consistency). The software Statistica 13.0 was used for the calculations.

## 3. Results and Discussion

### 3.1. Nutrients and Other Dietary Components from Hemp Cake

#### 3.1.1. Key Nutrients

Parametric values of DF, TOT Carb, and Dig Carb as well as EV were stable from 1 to 6 months after extraction but varied significantly depending on the technology used to obtain oil (Table 2). Only the water content remained at the same level and averaged 9.4% of the mass. The present study demonstrates that both dehulling and pressing conditions significantly influence the nutritional profile and energy value of hemp seed cake (HC). Dehulled hemp cake (DHC) exhibited a lower DF content (6.90–8.03%) compared to hulled hemp cake (HHC) (37.2–41.26%), yet was characterized by higher EV (512–540 kcal/100 g), likely due to increased lipid and available carbohydrate contents. These findings emphasize the functional role of seed coat removal in modulating the macronutrient balance of hemp-based products.

A comparable trend was not observed in the study by Mendoza-Pérez et al. [28], who analyzed defatted cake obtained from whole (hulled) seeds of three industrial hemp cultivars from Spain. Their results showed fiber contents between 34.5% and 38.8%, similar to our HHC values, reinforcing the notion that the seed coat is the principal fiber-contributing fraction. However, since no dehulling was applied in their protocol, differences due to seed coat removal could not be examined. Moreover, energy values were not directly reported, which limits direct comparison.

Carbohydrates in hemp seed by-products primarily consist of dietary fiber, both soluble and insoluble. The TOT Carb content in HHC ranged between 43.5% and 48.2%, whereas in DHC, it was only about 9.5%, indicating a more than four-fold difference. Similarly, Dig Carb was significantly higher in HHC (approx. 6.5%) than in DHC (approx. 2.3%) (Table 2). This suggests that the seed hull not only contains a substantial portion of indigestible polysaccharides but also contributes to the retention of other carbohydrate forms—likely associated with cell wall structures and residual starch.

These values are consistent with data reported by Leonard et al. [16], who stated that hemp by-products from hulled seeds typically contain 30–40% fiber, whereas dehulled seed products drop to 7–15%, which supports the validity of our findings. Moreover, they reported TOT Carb values ranging from 10 to 30%, predominantly as fiber. High dietary fiber levels, especially in hulled variants, support digestive health and can be used to develop high-fiber food products. From a practical perspective, HHC appears more suitable for applications requiring a high fiber content (e.g., functional foods, animal feed), while DHC—with its lower fiber and higher energy density—may serve better in high-calorie diets or products requiring enhanced bioavailability.

#### 3.1.2. Fatty Acid Profile

In terms of fatty acid composition, our results showed that the SFA content was slightly higher in HHC (10.73–11.21%), while MUFA dominated in DHC (11.0–14.2%), particularly following cold pressing (Table 3). This pattern is consistent with Leonard et al. [16], who reported that the mechanical pressing process influences the extraction efficiency of different lipid fractions, with MUFAs being more abundant in pressed cake due to their lower volatility and binding affinity to the solid phase of the oilseed cake. However, our study further suggests that the pressing temperature (cold vs. hot) has a measurable impact on the residual oil content and, consequently, the lipid profile of the cake, an aspect that Leonard et al. only briefly acknowledged. The n-6:n-3 fatty acid ratio in our samples (3.1–3.3:1) aligns with the values recommended for human nutrition (≤4:1), and closely matches results presented by Wang and Xiong [39] and Karabulut et al. [8], who emphasized the nutraceutical potential of hemp seed-based products due to their favorable PUFA composition. These ratios were consistent across DHC and HHC, indicating that seed dehulling and pressing do not significantly alter the essential fatty acid profile, but rather the total lipid yield [16].

The fatty acid profile in ‘Henola’ HC is highly consistent with that reported by Mendoza-Pérez et al. [28] in ‘Henola’ oil (solvent and SSP extraction). Our study showed that PUFA in HC amounted to 63–72%, while in the Spanish study, PUFA in oil amounted to 72.4–73.2%. The slightly lower level in our HHC/DHC was likely due to residual oil levels. MUFA in HC reached 10.1–14.0%, and additionally DHC reached comparable MUFA levels to the results obtained in oil (14.0–14.6%). Similar in magnitude, the SFA contents for ‘Henola’ oil (10.0–10.2%) and ‘Henola’ HC (7.5–11.2%) were accompanied by a significantly lower SFA content in our DHC (7.5–8.8%).

The results confirm that cold-pressed hemp cake retains a favorable unsaturated fatty acid profile, closely resembling that of pressed oils. The PUFA content remains high in both cake and oil, highlighting the value of hemp cake as a functional by-product. The ω-6/ω-3 ratio is within the optimal nutritional range, supporting the cardiovascular benefits of hemp-derived lipids.

#### 3.1.3. Other Food Components

Significantly higher contents of OC, CP, and sugars were attributed to DHC, which ranged between 33.1% and 37.5% for OC, 41.15% and 42.20% for CP, and 4.70% and 4.83% for sugars, respectively (Table 4). With HHC, these contents were higher on average by 27.0, 11.3, and 0.36% points. According to several studies, the residual OC in raw hemp seed oil cake ranges from 7% to 15%, depending on the pressing method and variety [5,7,40,41].

Residual OC in HC is a key parameter influenced by the efficiency of mechanical pressing and the presence of hulls. In one of the earliest reports, Callaway [5] noted that cold-pressed hemp cake typically retains between 7% and 10% oil, depending on extraction precision. Later, House et al. [11] analyzed several commercially available hemp seed-derived products and found oil contents ranging from 8% to 12%, highlighting the effect of the pressing method and seed variety. Galasso et al. [41] reported slightly higher values (10–15%), particularly in cakes derived from cold pressing with a lower mechanical yield, emphasizing the limitations of this technique. These findings were further corroborated by Leonard et al. [40], who cited similar residual oil ranges across various raw hemp seed cake samples. Our results for HHC and DHC are in agreement with these previous studies, particularly in the case of HHC (7.5–9.5% OC), which falls within the commonly reported range for cold-pressed cakes. Additionally, these values are consistent with the findings from a recent study on the ‘Henola’ cultivar [28], where defatted cake produced by screw-press extraction retained approximately 10.7% residual oil. These findings confirm that cold pressing, even under optimized mechanical conditions, typically leaves a substantial fraction of oil in the cake matrix, contributing to its energy value and potential functional applications.

The CP and CF contents in our samples were strongly influenced by the presence or absence of the seed hull. In the HHC, the CP content ranged from 28.4% to 31.4%, while CF ranged from 31.3% to 32.8%. These values are in strong agreement with earlier reports [5,11,39], where raw or minimally processed hemp seed oil cake typically contained 25–35% CP and 27–33% CF. A recent study [28] also reported comparable protein (~31.5%) and fiber (~28–30%) levels in defatted cakes derived from cold pressing whole seeds of ‘Henola’. Our findings are further corroborated by more recent studies. Shen et al. [42] demonstrated that proteins extracted from dehulled industrial hemp seeds exhibited significantly higher protein purity (up to 93.3%) due to the removal of hull components, affirming the role of hull removal in increasing the protein concentration and nutritional quality of hemp seed products. Their work also emphasized that adjusting precipitation pH during protein extraction substantially impacts the yield and purity of hemp protein isolates, aligning with our observations regarding the benefits of seed dehulling and precise processing conditions. Additionally, Tufarelli et al. [43] reported positive effects of incorporating hemp seed cake (HSC) into poultry diets, highlighting improved fatty acid profiles and oxidative stability in meat, owing to the rich nutritional profile of hemp components. This study not only underscores the nutritional benefits of hemp seed cakes but also reinforces the economic and environmental viability of their use as functional ingredients in animal feed. Similarly, Papatzimos and Kasapidou [44] comprehensively reviewed the utilization of hemp components, confirming that hemp cake, rich in proteins and essential fatty acids, significantly contributes to improved nutritional quality in animal products. Their findings, emphasizing enhanced polyunsaturated fatty acid (PUFA) levels and improved sensory attributes in products from animals fed with hemp supplements, complement and support our results. Further supporting these conclusions, Kasula et al. [45] evaluated the nutritional and safety profiles of hemp seed cake, affirming its suitability as a safe, nutrient-rich ingredient for animal feeds, which can effectively replace traditional protein sources and significantly enhance animal health outcomes. Similarly, Dong et al. [46] highlighted the versatility and efficacy of hemp seed proteins in food applications, noting their excellent functional properties, such as emulsification, foaming, and water-binding capacity, which are crucial for both animal nutrition and human food industries. Together, these studies consistently indicate that hemp seed processing methods, particularly dehulling and defatting, substantially enhance protein purity and nutritional value, meaning hemp seed cake is a highly beneficial ingredient in animal nutrition and functional food applications.

### 3.2. Content of Nutrients and Antinutritional Substances

#### 3.2.1. Indigestible Carbohydrates and Phytates

Single and double cold pressing (55 °C) of DHC, as well as hot pressing (90 °C), significantly reduced the levels of total non-starch polysaccharides (T-NSPs) and phytic acid (UA) when compared to the single cold pressing of HHC (Table 5). Considering the additional energy input required for heating or double pressing, the single cold pressing method appears to be both effective and more energy-efficient, while still reducing the antinutritional load to a safe level. Among all tested variants, hot-pressed DHC showed the highest protein content (59.72%) and ash (9.46%) but the lowest oil content (20.90%). In contrast, cold-pressed HHC contained the highest concentrations of antinutritional substances, with T-NSP exceeding 27% and UA reaching 2.62% of dry matter. This supports the role of the seed hull as a major reservoir of indigestible carbohydrates and phytates.

Hemp seeds are known to contain several antinutritional compounds, among which phytic acid is the most prevalent, reaching levels of up to 22.5 mg/g in whole seeds [47]. Other constituents include lignin, tannins, and protease inhibitors, such as trypsin inhibitors, which have also been detected in hemp seed meal [5]. These substances are predominantly located in the cotyledons and seed coats. Phytates exert their antinutritional effect by chelating essential minerals, particularly iron (Fe) and zinc (Zn), thereby reducing their bioavailability and impairing protein digestibility [48].

From an industrial perspective, hemp seed oil cake is often processed and marketed as hemp protein meal (HPM) [49] and is increasingly used as a high-protein and energy-rich feed additive in animal nutrition. Recent studies, including Kasula et al. [45] and Dong et al. [46], have confirmed the safety and efficacy of hemp seed cake, highlighting its nutrient-rich composition and absence of significant antinutritional risks when adequately processed. Kasula et al. [45] specifically emphasized the safety profile of hemp seed cake, noting negligible levels of antinutritional factors like cannabinoids, mycotoxins, and heavy metals, which further supports its inclusion in balanced animal diets.

Additionally, bioactive phenolic compounds, such as those identified by Sieger et al. [50] in cold-pressed hemp oil, exhibit strong antioxidant properties. These compounds may partially counteract the effects of antinutritional substances like phytates, enhancing the overall nutritional and functional value of hemp-derived products.

While indigestible carbohydrates such as T-NSP may reduce nutrient availability, they are also considered functional dietary fibers in human diets, contributing positively to gut health and supporting microbiota activity [51]. In humans, daily phytic acid intakes of up to 1% of the total diet (250–500 mg/day) are considered safe. Importantly, none of the analyzed variants in this study exceeded this threshold, suggesting that hemp cake—including HHC—can be safely incorporated into balanced diets when consumed in moderation.

#### 3.2.2. Substances from Fiber Fractions

TPC in the tested HCs, expressed in mg of gallic acid equivalents (GAEs), ranged from 0.98 to 2.48 mg GAE/g dry matter (D.M.) (Table 6). These values are lower than the range reported by Chen et al. [52] (3.9–15.6 mg GAE/g D.M.) and fall within the upper range observed by Leonard et al. [53]. This suggests that while hemp cake retains a portion of phenolic compounds, their concentration is highly influenced by the oil extraction method and the presence or absence of the seed hull. Recent research highlights the growing interest in hemp seed-derived polyphenols and their associated biological activities [42,53].

Among fiber-associated compounds, lignin and phytates—once considered antinutritional—are now increasingly recognized for their antioxidant properties [43,54]. Specific phenolic amides such as lignanamides, including cannabisin A, C, D, and M, exhibit notable antioxidant activity in DPPH radical scavenging assays, comparable to that of quercetin [55]. Cannabisin B demonstrates free radical neutralization and induces autophagic cell death in HepG2 liver cells [56]. Additionally, cannabisin Q and several related lignanamides have shown inhibitory effects on TNF-α release from LPS-induced BV2 microglial cells, indicating possible neuroprotective potential against neurodegenerative disorders [57].

All DHC products tested in this study were characterized by lower TPC levels (<1.97 mg GAE/g D.M.) and insoluble dietary fiber contents (DF, 6.67–9.39%) compared to HHC, which contained 2.48 mg GAE/g D.M. TPC and 51.81% DF. A significant reduction in PA content was observed in both single and double cold-pressed DHC variants (1.08% and 1.16%) compared to hot-pressed DHC (1.41%) and HHC (1.59%). This decrease may suggest that phenolic acid degradation is more pronounced under cold pressing than at higher temperatures, potentially due to reduced matrix stability or shorter exposure to oxidative stress.

### 3.3. Storage Tests of Hemp By-Products

The results of this study indicate that HCs, due to their relatively high lipid content, are susceptible to both oxidative and hydrolytic degradation over time. A comparison between HHC and DHC variants revealed distinct degradation patterns: HHC displayed higher acid values (AVs), indicating accelerated fat hydrolysis, while DHC exhibited higher peroxide values (PVs), signaling increased primary lipid oxidation (Table 7). Both types of cakes maintained acceptable quality during the first 1–3 months post-extraction. However, by the 6th month, Totox index values exceeded 15 in both HHC and DHC, indicating significant quality deterioration and reduced suitability for human consumption. These values were notably higher than those typically observed in soy-based analogs, suggesting lower oxidative stability of hemp seed-derived by-products under comparable storage conditions. These findings are consistent with the conclusions drawn by Callaway [5], who emphasized the vulnerability of hemp seed lipids to oxidative degradation due to their high content of PUFAs. Furthermore, Siger et al. [50] confirmed that although cold-pressed hemp seed oil contains natural antioxidants, including phenolic compounds, their concentration may be insufficient to fully protect the oil from progressive oxidative degradation over time. In our study, this was particularly evident in DHC, where peroxide values were higher despite the absence of hull-derived fiber matrices that could serve as additional antioxidant reservoirs. From a practical perspective, HHC may be better suited to use in ruminant feeds, where slower oxidation and a higher fiber content are desirable. DHC, on the other hand, presents better sensory and nutritional quality for human consumption, especially when consumed within three months of production. However, its higher oxidative susceptibility necessitates the application of protective storage strategies, including vacuum sealing and the elimination of light exposure. These observations contribute to the development of storage guidelines for hemp-derived food ingredients, highlighting the critical balance between technological processing (dehulling, pressing), product composition, and oxidative stability. The presence of the seed hull in hemp cakes was shown to enhance oxidative stability by reducing the formation of primary oxidation products, as reflected by lower peroxide values (PVs).

However, it simultaneously promoted lipid hydrolysis, resulting in significantly elevated acid values (AVs). This trade-off is consistent with structural and compositional interactions described in food by-products with high fiber contents. These observations are supported by the findings of Leonard et al. [58], who emphasized that the physical architecture of fiber-rich plant by-products, including hulls, can act as both protective barriers against oxygen diffusion and sites of accelerated lipid breakdown due to retained water and endogenous enzymes.

### 3.4. Storage Test of Hemp Oil

The results of this study revealed that hemp oil (cv. ‘Henola’) is highly susceptible to chemical degradation during storage. A sharp increase in AV was observed after three months—from 7.52 to 16.80 mg/g KOH—indicating the release of free fatty acids and the progression of lipid hydrolysis (Table 8). This increase corresponded to a nine-fold rise in free fatty acid (FFA) content, from 0.90% to 8.40%, expressed as oleic acid. Interestingly, the PV, which indicates primary oxidation products, initially decreased slightly between month one and month three (from 2.28 to 1.80 meq O_2_/kg). This may be attributed to the decomposition of hydroperoxides into secondary oxidation products—a well-documented phenomenon in oil storage studies. By the sixth month, however, PV doubled to 4.89 meq O_2_/kg, clearly indicating renewed oxidative activity and the breakdown of fatty acid structures. Overall, the Totox index, a composite indicator of total oxidation (TI = 2PV + ANV), rose from 5.05 in fresh oil to 10.73 after six months, exceeding the acceptable threshold for oil quality and confirming that the product had lost oxidative stability (Table 8).

Vacuum oven drying (VOD) is an economical method with adjustable drying parameters to avoid protein denaturation and loss of bioactive components [59]. The effects of the drying method on physicochemical, functional, and nutritional properties of HM-PI were investigated as a first step on the potential of HM-PI as a food ingredient. These findings are consistent with previous studies that have demonstrated the limited shelf life of cold-pressed hemp seed oil, particularly due to its high content of PUFAs, which are prone to both hydrolytic and oxidative degradation. Their study highlighted that mild drying conditions (e.g., low-temperature convection or VOD) preserved lipid integrity and minimized free fatty acid release, whereas more aggressive treatments resulted in increased acid values and peroxide accumulation, both of which are consistent with the degradation patterns observed in our stored hemp oil samples. Debedas et al. [60] also emphasize that cold pressed oils are quite susceptible to oxidation, easily initiated by factors such as light and temperature. During processing and storage as food, the oxidative stability of such oils is of great importance to ensure that the final product is healthy and safe throughout its shelf life. The current work, together with previous findings, reinforces the notion that hemp seed-derived lipid fractions are highly sensitive to environmental and processing stress. As such, maintaining low-oxygen, low-temperature storage conditions—in combination with optimized post-extraction handling—is critical to preserving product quality and extending the shelf life [61]. Given the observed instability after three months, hemp oil should be marketed and consumed promptly after extraction, and product labeling should clearly reflect expiration timelines and storage recommendations [62].

### 3.5. Microbiological Status of Hemp By-Products

The microbiological status of hemp seed cakes was evaluated over a six-month storage period to assess hygienic safety and potential shelf-life implications. Overall, the results indicate that the microbial quality of both HHCs and DHCs remained within acceptable safety limits during storage (Table 9). In HHC, a gradual increase in the total count of MAM was observed—from <100 cfu/g at baseline to 9980 cfu/g after six months. A similar trend was noted for Y&M, which reached 279 cfu/g. This increase is likely attributed to residual fungal spores and aerobic bacteria colonizing the outer hull, which may act as a microenvironment favorable to microbial persistence.

Despite the moderate microbial growth in HHC, all measured values remained below internationally accepted safety thresholds. No coliforms, coagulase-positive staphylococci (CPS), or *Enterobacteriaceae* were detected at levels above the detection limit at any time point, and *Salmonella* spp. was not detected in any sample. In contrast, DHC products exhibited excellent microbiological stability. Across the entire storage period, MAM counts remained consistently low (≤1000 cfu/g), and Y&M counts did not exceed 100 cfu/g. No indicator or pathogenic bacteria were detected in DHC samples. The microbiological stability of food industry by-products such as oilcakes is dependent on many factors, including moisture content, storage conditions, and the presence of natural antibacterial compounds. Although specific research on hemp oilcakes is limited, analyses of other cold-pressed oils suggest that the presence of natural antioxidants may also contribute to inhibiting the growth of some microorganisms [61]. Although data for hemp cakes are limited, Parry et al. [63] showed that the phenolic content of berry seed oils affects not only antioxidant properties but also microbial growth inhibition. Analogous mechanisms may occur in hemp cakes, especially when they contain residual amounts of fat rich in bioactive compounds.

### 3.6. Sensorial Analysis

The sensorial quality of hemp seed cakes was evaluated at 1, 3, and 6 months of storage using a trained three-member panel under standardized laboratory conditions. The evaluated parameters included general appearance, consistency, color, and odor. Across all time points, both HHC and DHC maintained favorable and stable organoleptic properties (Table 10). Both product types exhibited a loose, free-flowing texture, with no evidence of caking or clumping. Color remained stable throughout storage: HHC retained a grayish-brown hue, while DHC maintained a lighter beige tone. In terms of smell, DHC was perceived as more aromatic and herbaceous, while HHC exhibited a milder, plant-based aroma. These results support the conclusion that hemp cake is organoleptically stable over six months under appropriate storage conditions.

Studies on cold-pressed oils indicate that the presence of natural antioxidants can affect the sensory profile of the product, giving it a characteristic aroma and taste. However, a high content of polyunsaturated fatty acids can lead to a faster deterioration of sensory characteristics due to oxidation processes [64]. Similarly, Karamać et al. [65] found that hulls in oilseed residues can help delay sensory degradation by contributing phenolic compounds with antioxidant activity. Moreover, Matthäus and Brühl [66] emphasized that cold-pressed oilseed co-products retain their natural aroma and taste when stored away from light and oxygen—conditions that were met in this study. The fact that both HHC and DHC maintained a consistent sensory quality over time further supports their application as functional and shelf-stable food ingredients. From a microbiological perspective, no spoilage-related odors or discoloration were detected in any of the samples, which aligns with the results of the microbial assays and reinforces the sensory findings.

## 4. Conclusions

This study provides the first comprehensive evaluation of the long-term storage stability and nutritional dynamics of dehulled and hulled hemp cakes derived from the ‘Henola’ variety under real storage conditions. The comparison of processing variants revealed significant differences in protein content, fiber levels, oxidative resistance, and microbial safety. Dehulled hemp cake (DHC) proved especially promising due to its high nutritional density and superior shelf-life characteristics. The results provide a scientific basis for the development of hemp-based food supplements or animal feed formulations, particularly where shelf stability and nutritional density are crucial. This study highlights the significant impact of seed dehulling and processing conditions on the nutritional and functional value of hemp seed cake. Dehulled hemp cake (DHC) demonstrated superior protein and oil contents, reduced fiber and antinutritional compounds, and greater microbiological stability during storage. In contrast, hulled hemp cake (HHC) offered a higher dietary fiber content and slower primary lipid oxidation, suggesting its potential use in high-fiber food or feed products. Both types of hemp cake maintained organoleptic stability over a six-month period, supporting their suitability for shelf-stable formulations. Given the favorable fatty acid profiles, safe microbial status, and versatile macronutrient composition, cold-pressed hemp seed cake—particularly DHC—represents a valuable co-product for the development of functional foods, high-protein formulations, or feed supplements. Further research should explore its bioactive compound retention and consumer acceptability in final products.

## 5. Patents

[P] A. Wenda-Piesik., K. Ambroziak. ‘Method of producing protein-energy products based on oil seeds derived from soybean and hemp seeds’. The application was numbered: P.450940.

## Figures and Tables

**Table 1:** Functional features of the ‘Henola’ variety.

Trait	Value
Vegetation	100 days
Oil content	28–32%
Crude protein	20–22%
Crude fiber	30–32%
Yield	0.8–2.0 t ha^−1^
TSW	16–18 g

https://programkonopny.pl/program-konopny---henola.html, available date 14 November 2024.

**Table 2:** Nutritional components values (mean ± *s_e_*) of hemp cake after cold extraction of the oil from the seeds, cv. ‘Henola’.

HC	Length of Trial (Month)	DF (%)	TOT Carb (%)	Dig Carb (%)	EV (kcal 100 g^−1^)	Water (%)
HHC	1	37.20 ± 1.17 ^A 1^	47.30 ± 1.10 ^A^	6.30 ± 0.34 ^A^	287.00 ± 1.65 ^B^	10.50 ± 0.09
	3	41.26 ± 0.42 ^A^	48.15 ± 1.25 ^A^	6.89 ± 0.91 ^A^	292.50 ± 2.83 ^B^	9.74 ± 0.06
	6	37.65 ± 0.24 ^A^	43.46 ± 0.33 ^A^	6.13 ± 0.86 ^A^	305.50 ± 3.33 ^B^	8.90 ± 0.14
DHC	1	8.03 ± 0.79 ^B^	9.46 ± 0.17 ^B^	1.88 ± 0.75 ^B^	525.00 ± 3.30 ^A^	9.66 ± 0.16
	3	7.20 ± 0.59 ^B^	9.50 ± 0.10 ^B^	2.80 ± 0.43 ^B^	540.00 ± 6.60 ^A^	9.50 ± 0.31
	6	6.90 ± 0.10 ^B^	9.70 ± 0.02 ^B^	2.20 ± 0.10 ^B^	512.00 ± 4.71 ^A^	9.20 ± 0.05

^1^ Capital letters indicate significant differences between HHC and DHC at the same length of trial according to the *t*-Student test, at *p* = 0.05.

**Table 3:** Content values (mean ± *s_e_*) of fatty acids in hemp cake after cold extraction of the oil from the seeds, cv. ‘Henola’.

HC	Length of Trial (Month)	SFA (%)	MUFA (%)	PUFA (%)	n-3 (%)	n-6 (%)
HHC	1	10.90 ± 0.01 ^A 1^	10.13 ± 0.75 ^B^	62.30 ± 2.13	15.00 ± 0.53	45.00 ± 1.74
	3	10.73 ± 0.59 ^A^	13.03 ± 0.41 ^A^	72.26 ± 1.84	17.17 ± 0.26	52.40 ± 1.16
	6	11.21 ± 0.65 ^A^	10.92 ± 0.20 ^B^	63.26 ± 2.51	14.95 ± 0.68	47.16 ± 1.61
DHC	1	7.46 ± 0.78 ^B^	14.02 ± 0.07 ^A^	71.48 ± 0.14	14.16 ± 0.12	54.72 ± 0.10
	3	8.80 ± 0.48 ^B^	11.00 ± 0.47 ^B^	67.01 ± 0.46	16.90 ± 0.14	50.00 ± 0.24
	6	8.70 ± 0.50 ^B^	13.00 ± 0.24 ^A^	65.05 ± 0.34	16.30 ± 0.02	49.00 ± 0.21

^1^ Capital letters indicate significant differences between HHC and DHC at the same length of trial according to the *t*-Student test, at *p* = 0.05.

**Table 4:** Content values (mean ± *s_e_*) of the remaining food components from hemp cake following the cold extraction of oil from the seeds, cv. ‘Henola’.

HC	Length of Trial (Month)	OC (%)	CP (%)	Ash (%)	Sugar (%)	CF (%)
HHC	1	8.90 ± 0.10 ^B 1^	31.40 ± 0.71 ^B^	5.60 ± 0.10	2.90 ± 0.03 ^B^	32.40 ± 0.26 ^A^
	3	7.48 ± 0.76 ^B^	28.39 ± 0.36 ^B^	6.09 ± 0.06	3.07 ± 0.02 ^B^	31.35 ± 0.75 ^A^
	6	9.47 ± 0.28 ^B^	30.76 ± 0.29 ^B^	6.42 ± 0.23	3.22 ± 0.06 ^B^	32.75 ± 1.10 ^A^
DHC	1	37.50 ± 0.19 ^A^	41.15 ± 0.09 ^A^	6.75 ± 0.01	4.83 ± 0.04 ^A^	2.00 ± 0.09 ^B^
	3	35.20 ± 0.49 ^A^	41.40 ± 0.19 ^A^	6.50 ± 0.05	4.80 ± 0.02 ^A^	1.90 ± 0.01 ^B^
	6	33.10 ± 0.38 ^A^	42.20 ± 0.07 ^A^	6.30 ± 0.02	4.70 ± 0.02 ^A^	1.90 ± 0.02 ^B^

^1^ Capital letters indicate significant differences between HHC and DHC at the same length of trial according to the *t*-Student test, at *p* = 0.05.

**Table 5:** Content values (mean ± *s_e_*) of nutritional and antinutritional substances in dry matter of hemp cake, cv. ‘Henola’, depending on the oil extraction process.

Oil Extraction Process of HC	Content (%) D.M.				
	Protein	Oil	Ash	T-NSP	UA
1 cold pressing (55 °C) of DHC	43.33 ± 0.06 ^b 1^	43.93 ± 0.00 ^c^	6.94 ± 0.02 ^a^	3.91 ± 0.03 ^a^	1.19 ± 0.01 ^a^
2 cold pressing (55 °C) of DHC	42.35 ± 0.04 ^b^	42.37 ± 0.00 ^c^	6.90 ± 0.01 ^a^	2.70 ± 0.05 ^a^	0.81 ± 0.03 ^a^
1 hot pressing (90 °C) of DHC	59.72 ± 0.23 ^c^	20.90 ± 0.02 ^b^	9.46 ± 0.03 ^b^	3.22 ± 0.04 ^a^	1.10 ± 0.02 ^a^
1 cold pressing (55 °C) of HHC	29.95 ± 0.36 ^a^	15.94 ± 0.02 ^a^	6.75 ± 0.03 ^a^	27.94 ± 0.48 ^c^	2.62 ± 0.00 ^b^

^1^ Small letters indicate significant difference between processes according to Tukey’s HSD test, at ±0.05.

**Table 6:** Content of antinutritional substances from fiber fraction in dry matter of hemp cake, cv. ‘Henola’, depending on the oil extraction process.

Oil Extraction Process of HC	Content (%) D.M.				
	KL	RFO	DF	PA	TPC ^1^
1 cold pressing (55 °C) of DHC	2.80 ± 0.03 ^a 2^	1.49 ± 0.00 ^b^	9.39 ± 0.02 ^a^	1.08 ± 0.01 ^a^	1.96 ± 0.00 ^ab^
2 cold pressing (55 °C) of DHC	1.99 ± 0.03 ^a^	1.18 ± 0.00 ^b^	6.67 ± 0.01 ^a^	1.16 ± 0.00 ^a^	0.98 ± 0.01 ^a^
1 hot pressing (90 °C) of DHC	2.26 ± 0.01 ^a^	1.78 ± 0.02 ^b^	8.37 ± 0.07 ^a^	1.41 ± 0.01 ^b^	1.31 ± 0.02 ^a^
1 cold pressing (55 °C) of HHC	20.62 ± 0.21 ^b^	0.63 ± 0.02 ^a^	51.81 ± 0.24 ^b^	1.59 ± 0.01 ^b^	2.48 ± 0.02 ^b^

^1^ mg GAE g^−1^ D.M. ^2^ Small letters indicate significant difference between processes according to Tukey’s HSD test, at *p* = 0.05.

**Table 7:** Storage parameters of hemp cakes made from seeds, cv. ‘Henola’, depending on the time after the oil extraction process.

HC	Length of Trial (Month)	AV (mg g^−1^ KOH)	PV (meq O_2_ kg^−1^)	ANV	Totox Index
HHC	1	8.90 ^A 1^	0.10 ^A^	3.43 ^B^	3.63 ^A^
	3	21.93 ^B^	0.66 ^A^	6.00 ^B^	7.32 ^A^
	6	80.67 ^B^	4.12 ^A^	6.98 ^A^	15.21 ^A^
DHC	1	9.95 ^A^	1.20 ^A^	0.40 ^A^	2.80 ^A^
	3	17.70 ^A^	5.75 ^B^	0.82 ^A^	12.32 ^B^
	6	58.00 ^A^	6.79 ^A^	5.54 ^A^	19.12 ^B^

^1^ Capital letters indicate significant differences between HHC and DHC at the same length of trial according to the *t*-Student test, at *p* = 0.05.

**Table 8:** Storage parameters of hemp oil, ‘Henola’ variety, and total degree of product oxidation.

Length of Trial (Month)	AV (mg g^−1^ KOH)	FFA (%)	PV (meq O_2_ kg^−1^ Oil)	ANV	Totox Index
1	7.52 ^a 1^	0.90 ^a^	2.28 ^a^	0.50 ^a^	5.05 ^a^
3	16.80 ^b^	8.40 ^b^	1.80 ^a^	0.90 ^a^	4.50 ^a^
6	19.22 ^b^	1.60 ^a^	4.89 ^b^	0.95 ^a^	10.73 ^b^

^1^ Small letters indicate significant differences between months for oil trials according to the HSD-Tukey test, at *p* = 0.05.

**Table 9:** Colony forming unit (cfu g^−1^) increase in microbiological activity in hemp cakes.

HC	Month	MAM at 30 °C	*Coli*	Y&M	*Enterobacteriaceae*	CPS	*Salmonella*
HHC	1	<100	<10	<10	<10	<10	nd ^1^
	3	240	<10	<100	<10	<10	Nd
	6	9980	<100	279	<50	<10	Nd
DHC	1	<100	<10	<10	<10	<10	Nd
	3	<100	<10	<100	<10	<10	Nd
	6	1000	<10	<100	<10	<10	Nd

^1^ Not detected.

**Table 10:** Sensorial characteristics of hemp cakes during storage.

HC	Month	General Appearance	Consistency	Color	Smell
HHC	1	Clean, uniform, free of contamination	Dry, loose, non-caking	Grayish-brown	Mild, plant-based
	3	No visual change	Stable	Stable	Slight fading of aroma
	6	No visual change	Stable	Stable	Still acceptable, low intensity
DHC	1	Clean, uniform, no visible contamination	Dry, loose, non-caking	Beige	Fresh, aromatic, herbaceous
	3	No visual change	Stable	Stable	Maintained strong aroma
	6	No visual change	Stable	Stable	Aromatic intensity preserved

## Data Availability

The data presented in this study are available on request from the corresponding author due to privacy.
